# Public Engagement and other Essential Requirements for Data Trusts, Data Repositories and Other Data Collaborations.

**DOI:** 10.23889/ijpds.v7i3.2105

**Published:** 2022-08-25

**Authors:** Alison Paprica, Kim McGrail, Monique Crichlow, Donna Curtis Maillet, Sarah Kesselring, Conrad Pow, Thomas Scarnecchia, Michael Schull

**Affiliations:** 1 University of Toronto; 2 University of British Columbia; 3 University of New Brunswick; 4 Population Data BC; 5 North York General Hospital; 6 Digital Aurora; 7 ICES

## Objectives

To test and refine a list of 12 minimum specification essential requirements (min specs) for data trusts, data repositories, and other data collaborations that had been generated and published by a team of 19 Canadians in 2020.

## Approach

We convened an international team of more than 50 people to discuss, test, and refine the 12 min specs. Twenty-three (23) organizations tested the min specs and five analysis sub-teams were formed to identify commonalities and differences in terms of how the min specs are being fulfilled, and ways to improve the min specs. In parallel, we worked with Canada’s CIO Strategy Council to develop the voluntary standard “CAN/CIOSC 100-7 Operating Model for Responsible Data Stewardship” based on the updated min specs.

## Results

The list of min specs increased from 12 to 15: one for Legal, five for Governance, four for Management, two for Data Users, and three for Stakeholder & Public Engagement. The main changes were the division of requirements that had initially been grouped together under Stakeholder & Public Engagement, one new Governance min spec focused on Indigenous data sovereignty, one new Management min spec focused on data documentation, and multiple changes to make the min specs more precise and directive. The CAN/CIOSC 100-7 standard is progressing through committees and approvals and on track to be finalized by summer 2022. To our knowledge, it will be the first standard that identifies public engagement as a requirement for data trusts, data repositories, or other data collaboratives.

## Conclusion

Including international team members in the testing and refinement of the min specs led to significant improvements. The process we used may also benefit other teams and organizations who are working to progress from frameworks and principles to practical guidance.

